# Meta-analysis of metabolic syndrome and its individual components with risk of atrial fibrillation in different populations

**DOI:** 10.1186/s12872-021-01858-1

**Published:** 2021-02-15

**Authors:** Ying Zheng, Zengshuo Xie, Jiayong Li, Chen Chen, Wenting Cai, Yugang Dong, Ruicong Xue, Chen Liu

**Affiliations:** 1 Department of Otorhinolaryngology-Head and Neck Surgery, The First Affiliated Hospital of Sun Yat-Sen University, Guangzhou, China; 2 Department of Cardiology, The First Affiliated Hospital of Sun Yat-Sen University, Guangzhou, 510080 China; 3 NHC Key Laboratory of Assisted Circulation (Sun Yat-Sen University), Guangzhou, 510080 China; 4 National-Guangdong Joint Engineering Laboratory for Diagnosis and Treatment of Vascular Diseases, Guangzhou, China

**Keywords:** Metabolic syndrome, Atrial fibrillation, Observational cohort study, Risk factor

## Abstract

**Background:**

Recent studies have reported the effects of metabolic syndrome (MetS) and its components on atrial fibrillation (AF), but the results remain controversial. Therefore, we performed a meta-analysis to evaluate the relationship between MetS and AF risk.

**Methods:**

Studies were searched from the Cochrane library, PubMed, and Embase databases through May 2020. Adjusted hazard ratios (HRs) and its corresponding 95% confidence intervals (CIs) were extracted and then pooled by using a random effects model.

**Results:**

A total of 6 observational cohort studies were finally included. In the pooled analysis, MetS was associated with an increased risk of AF (HR 1.57; 95% CI 1.40–1.77; *P* < 0.01). And the components of MetS including abdominal obesity (HR 1.37; 95% CI 1.36–1.38; *P* < 0.01), elevated blood pressure (HR 1.56; 95% CI 1.46–1.66; *P* < 0.01), elevated fasting glucose (HR 1.18; 95% CI 1.15–1.21; *P* < 0.01) and low high density cholesterol (HDL) (HR 1.18; 95% CI 1.06–1.32; *P* < 0.01) was also associated with an increased risk of AF, while high triglyceride (HR 0.99; 95% CI 0.87–1.11, *P* = 0.82) was not.

**Conclusions:**

Our present meta-analysis suggested that MetS, as well as its components including abdominal obesity, elevated blood pressure, elevated fasting glucose and low HDL cholesterol were associated with an increase in the risk of AF.

## Background

Atrial fibrillation (AF) is the most common arrhythmia with an estimated 33.3 million affected people in the world in 2015 [1]. By 2050, the prevalence of AF in people over the age of 60 is estimated to increase from 3.9 million to 9 million [2]. AF is associated with a fivefold risk for stroke and causes 15% of all strokes [3]. In addition, AF is an independent risk factor for death, with a 1.5-fold risk for men and 1.9-fold risk for women for mortality after adjustment for known risk factors [4]. Therefore, it is important to investigate the etiology and influencing factors of AF.

Metabolic syndrome (MetS) represents a series of cardiovascular and metabolic disorders, defined as a cluster of central obesity, hypertension, dyslipidemia, and glucose intolerance [5]. MetS worsens vascular function and causes subclinical damage in multiple organs, which is a more serious single risk factor than traditional one [6]. Prior studies have found that the prevalence of AF increases in parallel with the frequency of MetS [7]. Epidemiological studies conducted in Asian population samples [8–11] and Western population samples [12, 13] showed that patients with MetS are more likely to have AF than patients without MetS. In addition, the increase in the number of components of the MetS is related to the occurrence of AF [14]. Despite the relatively large amount of evidence, there are differences in the estimates of the studies above, so we cannot assess the extent to which the MetS and its components affect AF [8–13]. Therefore, we conducted a systematic review and meta-analysis to observe the impact of MetS or its individual components on the incidence of AF.

## Methods

This meta-analysis was performed by following the Meta-Analysis of Observational Studies in Epidemiology (MOOSE) [15]. Meta-analysis of available articles does not require ethics approval.

### Definitions of MetS

The definitions of MetS adopted by the included studies in this article are not exactly the same, including National Cholesterol Education Program-Third Adult Treatment Panel (NCEP-ATPIII) [16], International Diabetes Federation [17] and American Heart Association and National Heart, Lung, and Blood Institute (AHA/NHLBI) [18]; and the study Nyström-2015 adopted other definition standards (see Additioanl file 1: Table 1).

### Literature search

We searched the original studies from the Cochrane library, PubMed, and Embase databases through May 2020. We combine the key words about ‘atrial fibrillation’ with the key words about ‘metabolic syndrome’ to search the relevant studies (see Additioanl file 1: Table 2). In addition, we also reviewed the references of the retrieved studies for further research. There was no language limitation used in the process.

### Eligibility criteria

Observational cohorts were adopted if they met all of the following criteria: (1) evaluating the effects of MetS and/or its components (abdominal obesity, elevated blood pressure, elevated fasting glucose, low high density cholesterol (HDL) and high triglyceride) on the risk of AF; (2) adjusted hazard ratios (HRs) and corresponding 95% confidence intervals (CIs) were regarded as the effect values. The exclusion criteria were as follows: (1) outcomes of the study were other AF-related diseases (e.g., AF recurrence, postoperative AF or atrial flutter); (2) certain publication types with no relevant data (e.g., reviews, letters, case reports, comments).

### Data extraction

The following data of all included articles was retrieved by Ying Zheng Ph.D. and Zengshuo Xie: author(s) name, publication year, country of original study, design of study, sample characteristics of study population (size, age range and gender composition), diagnostic criteria of MetS, outcome, and adjusted HRs with 95% CIs. If there were more than one HRs available in one study, the most fully adjusted one was used. Besides, in the cohort studies, we preferred to extract the risk estimates for the longest follow-up time. In addition, if one cohort was more than once published, we extracted the most recent one instead.

### Quality assessment

For the quality assessment, the selected cohort studies were evaluated by Newcastle–Ottawa scale (NOS). The quality of the studies was rated with a score from 0 to 9, representing the risk of bias from high to low [19]. Studies with a summary score above the median were considered to have low risk of bias.

### Statistical analysis

The primary results of this meta-analysis were focused on the prevalence of AF in people with MetS versus those without. In addition, the effects of individual components of MetS were also evaluated. Then, the effects were weighted against that calculated for MetS per se using the same datasets. I2 test was used to assessed Heterogeneity among the included studies (I2 ≥ 25%, I2 ≥ 50%, and I2 ≥ 75% were respectively defined as low, moderate, and high heterogeneity) [20]. The risk estimates and 95%CIs were accordingly transformed to natural logarithm (log HR) and standard error (SE). A random effects model was selected to calculate the summary results. It was reported that this model is more conservative than the fixed-effects models, and could provide better estimates with wider confidence intervals [21]. According to the Cochrane book, if < 10 studies are included, neither funnel plots nor statistical tests ([i.e., Begg test and Egger test) can be used meaningfully to test for publication bias.

All statistical analysis processes were executed by statistical software Revman Manager 5.3 (Nordic Cochrane Center; http://ims.cochrane.org/revman).

## Results

### Study selection

**Fig. 1:**
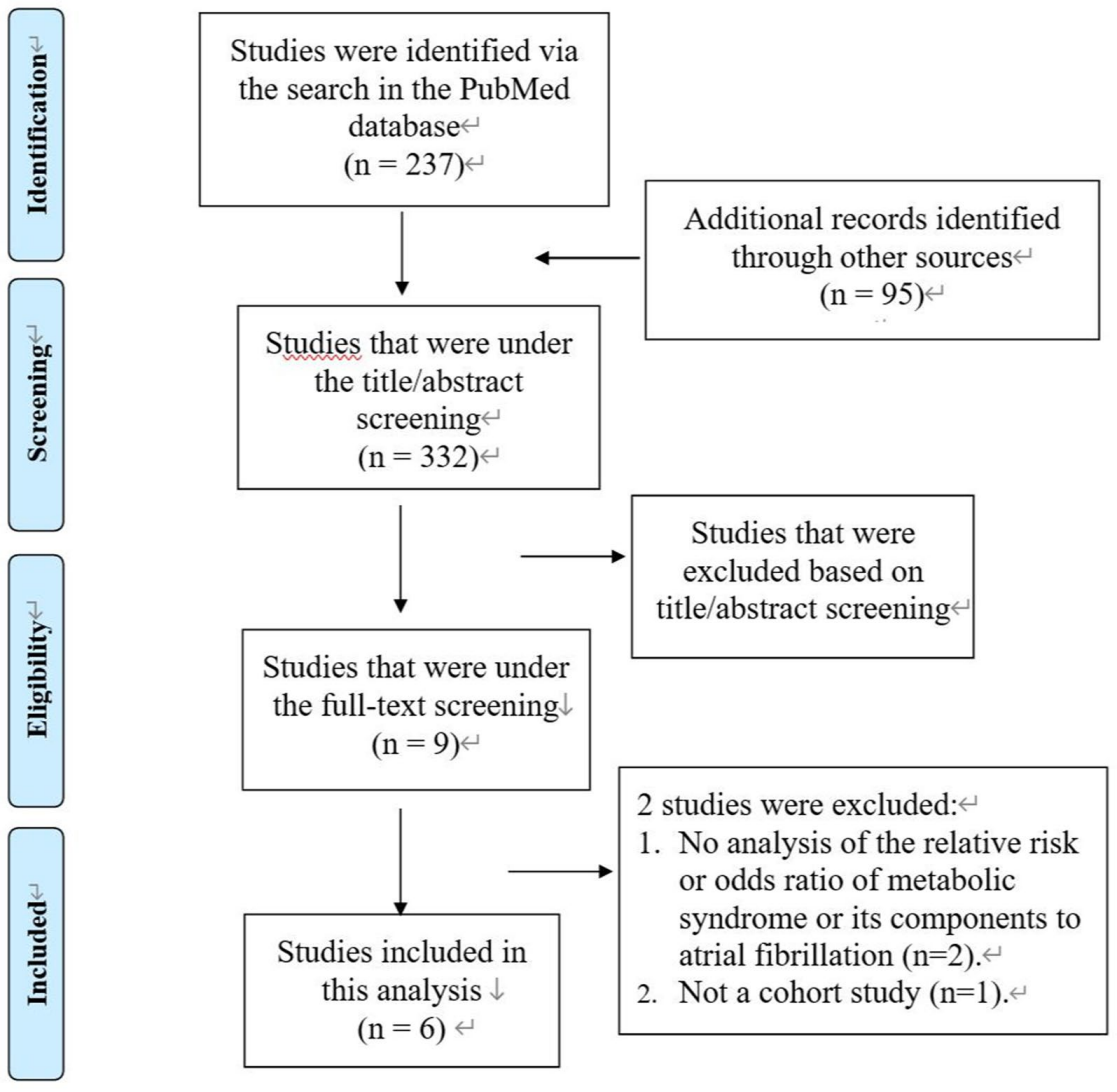
Study search diagram

Figure 1 shows the literature search process. Among 335 articles identified from the databases, we included 6 studies for analysis, including 4 prospective cohorts [8, 10, 12, 13] and 2 retrospective cohorts [9, 11], which described their results with HRs.

Table 1 summarizes the patients characteristics of the included studies. A total of 30,810,460 patients were analyzed, including 7,544,393 or 7,545,221 cases of MetS (with different definitions). The population included in the studies included Korean [9–11], Japanese [8], American [12] and Stockholmer [13]. Among them, one study [12] used the AHA/NHLBI criteria for the diagnosis of MetS and another one [10] used the International Diabetes Federation criteria. Two studies [9, 11] diagnosed the MetS according to the NCEP-ATPIII criteria. One study [8] conducted in Japan calculated the effects of MetS confirmed to NCEP-ATPIII and AHA/NHLBI criteria respectively. The last one study [13] used other definition.

**Table 1:** Summary of included studies in this meta-analysis

Studies	Study design	Total case (n)	Population	MetS rate	MetS definition	Follow-up, (years)	AF rate
Choe-2019	Retrospective cohort study	22,886,663	Korean (aged > 40 years) from the Korean National Health Insurance Service from 2008 to 2013	27.4%	NCEP-ATPIII	5.4	0.98%
Kwon-2019	Retrospective cohort study	7,830,602	Korean (aged 30–69 years) from the Korean National Health Insurance Service from 2009 to 2016	15.9%	NCEP-ATPIII	7.3	0.26%
Kim-2018	Prospective cohort study	21,981	Korean men from a health screening program from 2003 to 2008	11.5%	International Diabetes Federation	8.7	0.8%
Chamberlain-2010	Prospective cohort study	15,094	American (aged 45–64 years) from Atherosclerosis Risk in Communities study from 1987–1989	41.1%	AHA/NHLBI	15.4	8.2%
Nyström-2015	Prospective cohort study	4,021	Stockholmer (aged 60 years) from a health-screening study in Stockholm from August 1997 to March 1999	27.6%	Other definition standards	13.6	7.1%
Watanabe-2008	Prospective cohort study	28,449	Japanese (aged ≥ 20 years) from an annual health examination at the Niigata Association for Comprehensive Health Promotion and Research	13% for NCEP-ATPIII; 16% for AHA/NHLBI	NCEP-ATPIII or AHA/NHLBI	4.5	0.9%

Metabolic syndrome = MetS; National Cholesterol Education Program-Third Adult Treatment Panel = NCEP-ATPIII; American Heart Association and National Heart, Lung, and Blood Institute = AHA/NHLBI

The NOS scores of the included literatures are all greater than 6 and the qualities of them are acceptable.

### MetS with AF risk

**Fig. 2:**
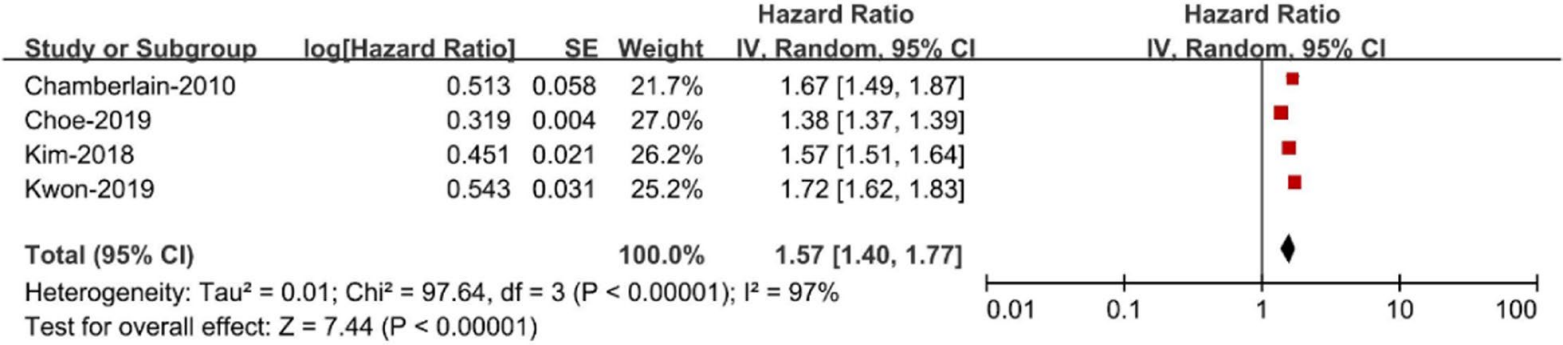
Metabolic syndrome and risk of atrial fibrillation

There were 4 studies [9–12] included in this part. The other 2 were excluded because Watanabe-2008 [8] defined obesity with Body Mass Index (BMI), instead of waist circumference (WC); Nyström-2015 [13] used normal weight, no MetS as the reference in statistics. Our meta-analysis showed that patients with MetS were at an increased risk of AF (RR 1.57; 95% CI 1.40–1.77; Fig. 2) as compared with those without MetS, with significant heterogeneity (I2 = 97%).

### Association of individual components of MetS with AF risk

**Fig. 3:**
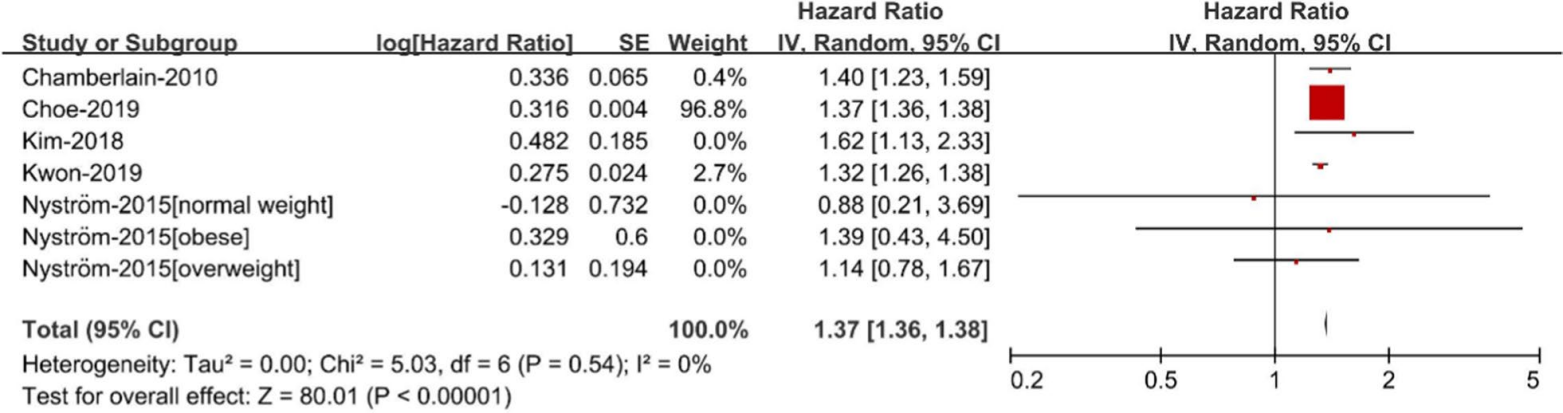
Risk of atrial fibrillation according to individual components of metabolic syndrome-Abdominal obesity

*Abdominal obesity* The risk of AF was elevated in patients with abdominal obesity as compared to those without abdominal obesity (RR 1.37; 95% CI 1.36–1.38, Fig. 3), with little heterogeneity (I2 = 0%). It is worth noting that after sorting by BMI, the risk of AF according to abdominal obesity was analyzed in various populations (normal; obese; overweight), and the results showed that abdominal obesity could not be proved to have a higher risk.

**Fig. 4:**
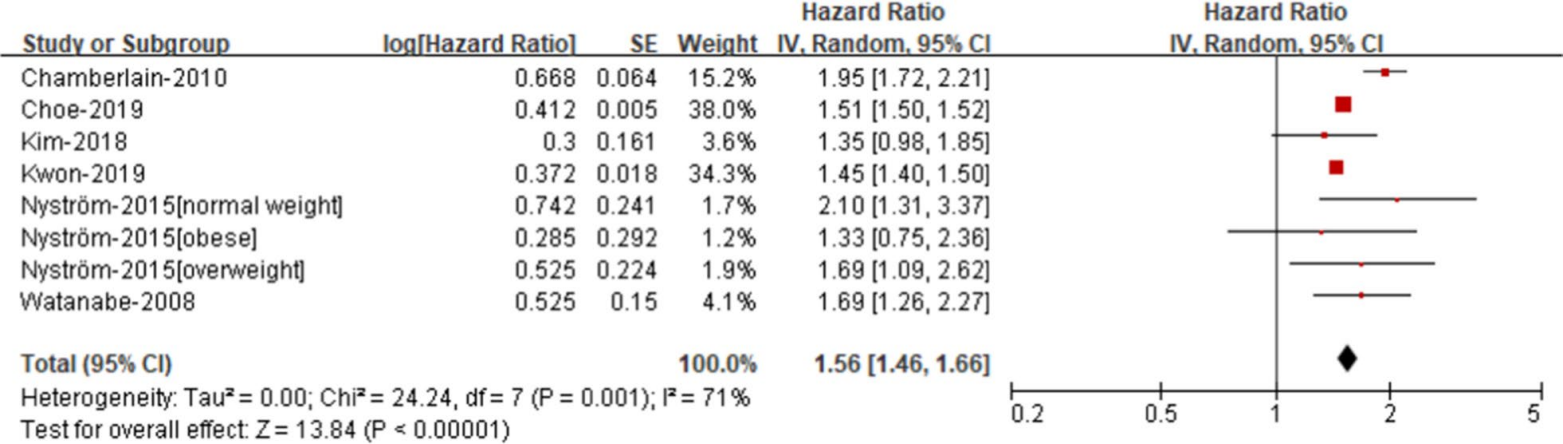
Risk of atrial fibrillation according to individual components of metabolic syndrome- Elevated blood pressure

*Elevated blood pressure* Our study showed that the risk of AF was elevated in patients with elevated blood pressure compare with those without elevated blood pressure (RR 1.56; 95% CI 1.46–1.66, Fig. 4), with moderate heterogeneity (I2 = 71%).

**Fig. 5:**
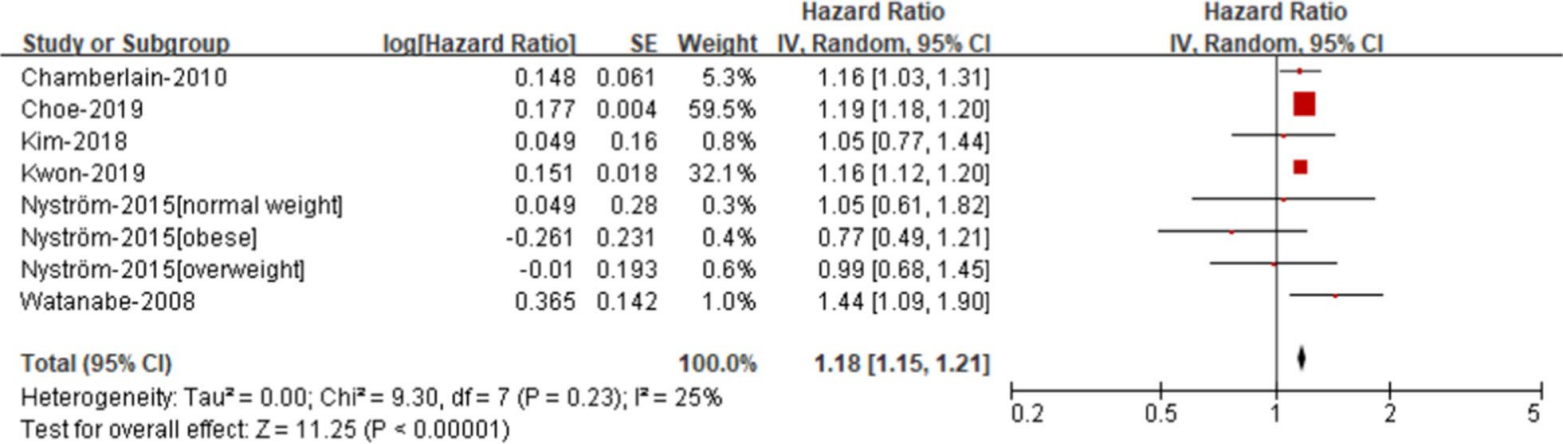
Risk of atrial fibrillation according to individual components of metabolic syndrome- Elevated fasting glucose

*Elevated fasting glucose* The risk of AF was elevated in patients with elevated fasting glucose compare with those without elevated fasting glucose (RR 1.18; 95% CI 1.15–1.21, Fig. 5), with low heterogeneity (I2 = 25%).

**Fig. 6:**
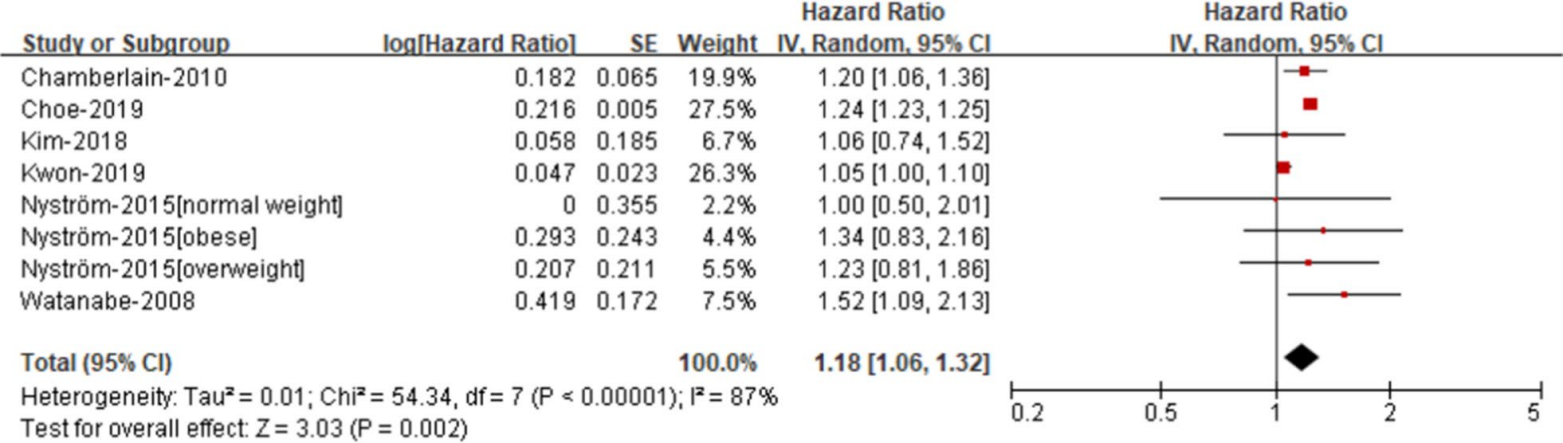
Risk of atrial fibrillation according to individual components of metabolic syndrome- Low HDL cholesterol

*Low HDL cholesterol* The risk of AF was elevated in patients with low HDL cholesterol compare with those without low HDL cholesterol (RR 1.18; 95% CI 1.06–1.32, Fig. 6), with significant heterogeneity (I2 = 87%).

**Fig. 7:**
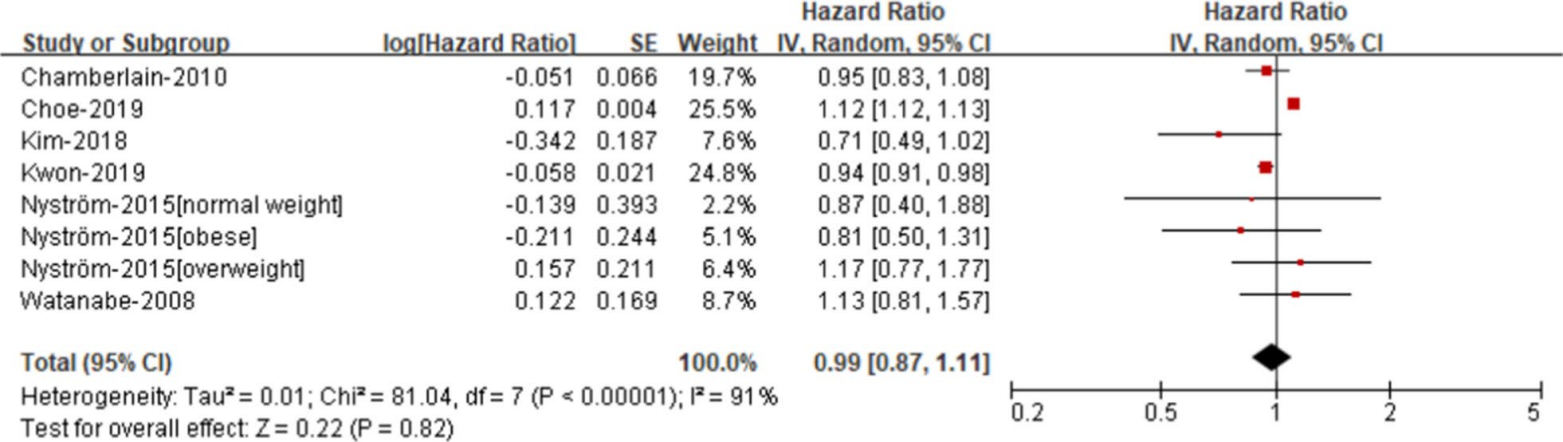
Risk of atrial fibrillation according to individual components of metabolic syndrome- High triglyceride

*High triglyceride* The risk of AF was not elevated in patients with high triglyceride compare with those without high triglyceride (RR 0.99; 95% CI 0.87–1.11, Fig. 7), with significant heterogeneity (I2 = 91%).

### Publication bias and sensitivity analysis

Since 6 studies evaluated the effects of MetS and its components on risks of AF, we did not examine the publication bias in this meta-analysis. In spite of this, the results were not changed in the sensitivity analysis as performed by removing the included studies one by one.

## Discussion

To our knowledge, this is the first meta-analysis of published studies from different regions to examine the relationship between MetS and AF risk. Our meta-analysis showed that patients with MetS were at an increased risk of AF as compared with those without MetS. For the components of MetS, abdominal obesity, elevated blood pressure, elevated fasting glucose and low HDL cholesterol were associated with an increase in the risk of AF.

### Comparison with other studies

There is no meta-analysis on the relationship between the risk of AF and MetS as well as its components. Many prior studies reported individual components of MetS as independent predictors of the risk of AF instead of evaluating all the MetS components [22–24]. Our result that MetS and its components can increase the risk of AF is roughly consistent with the results of previous clinical research. It is found that MetS is highly associated with paroxysmal AF and/or atrial flutter in patients without structural heart diseases [25]. Another study showed that in non-diabetic patients with non-essential hypertension, MetS is directly related to and independent of the prevalence of AF [14]. In addition, MetS also has an effect on AF outcome and recurrence risk catheter ablation [26, 27]. Our analysis showed a significant different result for the effect of triglycerides and AF risk as compared to other studies. In a prospective study, researchers found a higher risk of AF with elevated triglyceride levels [28]. This contradictory result may be related to the age of the population included in various studies. Research found that after 70 years of age, triglyceride levels will decline, and the prevalence of AF continues to increase with age [29]. Other possible reason could be the effect of advancing age with reduced circulating TSH levels and triglyceride levels [30, 31]. In epidemiological studies, this phenomenon may offset the effect of high triglyceride on the incidence of AF. Therefore, when analyzing the effect of high triglyceride on AF, it is necessary to group by age. Studies included in our analysis did not have specific age data thus limiting further interpretation of age and triglyceride effect. Therefore, we have concluded that high triglyceride will not affect AF.

### Implications and further research

Our study results show Mets and its components have significant influence on occurrence of AF, and the control of MetS and its components may have an effective effect on reducing the prevalence of AF.

Many studies have revealed the mechanism by which MetS increases AF risk. the possible cause of AF in MetS is the structural, functional and electrical changes of the left atrium. MetS causes increased inflammation and oxidative stress, which may lead to vascular dysfunction and makes MetS patients more prone to atrial remodeling [32, 33].

Among the components of MetS, hypertension and diabetes are two strong independent factors of AF [22, 23]. Experiments in a rat hypertension model show that hypertension can rapidly induce left atrium hypertrophy, fibrosis and inflammation, as well as electrophysiological changes, including shortened atrial wavelength and changes in Ca2 + current density [34]. These changes will increase the risk of AF. Insulin resistance leads to increased levels of C-reactive protein in the myocardium, which can induce myocardial fibrosis and diastolic dysfunction [33] as well as atrial dilation [35]. In addition, diabetes may induce autonomic nervous system dysfunction, thereby promoting the occurrence of AF [36]. Similarly, abdominal obesity is also associated with the increase the risk of AF [24]. Obesity not only directly causes MetS, but also increases the possibility of developing other MetS components [37]. Like hypertension and diabetes, obesity also promotes oxidative stress, inflammation, and myocardial fibrosis, which induces AF [33]. In addition, obesity is also closely related to obstructive sleep apnea which is associated with the prevalence of AF [37]. However, the mechanism of the effect of blood lipids on AF is still unclear. Researchers thought that this may be related to inflammation and oxidative stress, because the anti-inflammatory and antioxidant properties of HDL particles [38] can inhibit AF [33, 39].

### Strengths and limitations of study

This is the first meta-analysis on the relationship between the risk of AF and MetS as well as its components. But due to the limited number of studies, we need to acknowledge several limitations. Firstly of all, the research population of the three included studies is Korean, and in the meta-analysis, their total weight is more than 50%, especially in the analyses of abdominal obesity and elevated fasting glucose, which may be an important reason for bias. In addition, because MetS is completely different in Asian and Caucasian patients, it may also lead to uncertainty in the study results. Secondly, use of different definitions of Mets could have increased the heterogeneity seen. But if we only use studies with the same MetS definition, there may not be enough data for analysis. Unfortunately, we failed to explore the roots of heterogeneity. Finally, due to the nature of observational data included in this met-analysis, potential confounders cannot be ruled out, which may alter the final result.

## Conclusions

Our present meta-analysis suggested that MetS, as well as its components including abdominal obesity, elevated blood pressure, elevated fasting glucose and low HDL cholesterol were associated with an increase risk of AF.

## Supplementary Information

**Additioanl file 1.** The criteria of metabolic syndrome used in the included studies and the search strategies of this meta-analysis.

## Data Availability

The dataset used and analyzed in the current study can be obtained from Pubmed upon reasonable request. The datasets used and/or analysed during the current study available from the corresponding author of the included researches on reasonable request.

## Abbreviations

AF: atrial fibrillation
BMI: Body Mass Index
HDL: high density cholesterol
MetS: metabolic syndrome
NCEP-ATPIII: National Cholesterol Education Program-Third Adult Treatment Panel
RR: risk ratio
WC: waist circumference
AHA/NHLBI: American Heart Association and National Heart, Lung, and Blood Institute
CI: confidence interval
HR: hazard ratio
MOOSE: Meta-Analysis of Observational Studies in Epidemiology
NOS: Newcastle–Ottawa scale
SE: standard error
