# Supplementary material for: Meta-analysis of metabolic syndrome and its individual components with risk of atrial fibrillation in different populations
Source: BMC Cardiovasc Disord. 2021 Feb 15;21:90. doi: 10.1186/s12872-021-01858-1 (PMC7885417; doi:10.1186/s12872-021-01858-1)
Supplement: Supplementary file 1 — Additioanl file 1. The criteria of metabolic syndrome used in the included studies and the search strategies of this meta-analysis. [file 12872_2021_1858_MOESM1_ESM.docx]

Supplementary Table 1: The criteria of metabolic syndrome used in the included studies

| Studies | | MetS | Abdominal obesity | Elevated blood pressure | Elevated fasting glucose | High TG | Low HDL‐C |
| --- | --- | --- | --- | --- | --- | --- | --- |
| Choe-2019 | NCEP-ATPIII | | WC≥80 cm in women or ≥90 cm in men | SBP ≥130 mm Hg and / or DBP ≥85 mm Hg and / or a history of treated hypertension | fasting glucose≥100 mg/dL or treatment of previously diagnosed diabetes | triglycerides ≥150 mg / dL or drug treatment for high triglyceride | HDL‐C <40 mg/dL for men, <50 mg/dL for women or drug treatment for low HDL‐C |
| Kwon-2019 | | NCEP-ATPIII | WC ≥ 90 cm in men or ≥ 85 cm in women | SBP ≥130 mm Hg and / or DBP ≥85 mm Hg and / or a history of treated hypertension | fasting glucose≥100 mg/dL or treatment of previously diagnosed diabetes | triglycerides ≥150 mg / dL or drug treatment for high triglyceride | HDL‐C <40 mg/dL for men, <50 mg/dL for women or drug treatment for low HDL‐C |
| Kim-2018 | | International Diabetes Federation | WC ≥ 90 cm | SBP ≥130 mm Hg and / or DBP ≥85 mm Hg and / or a history of treated hypertension | fasting glucose≥100 mg/dL or treatment of previously diagnosed diabetes | triglycerides ≥150 mg / dL or drug treatment for high triglyceride | HDL‐C <40mg/dL or drug treatment for lowering HDL-C |
| Chamberlain-2010 | | AHA/NHLBI | WC≥88 cm in women or ≥102 cm in men | SBP ≥130 mm Hg and / or DBP ≥85 mm Hg and / or a history of treated hypertension | fasting glucose≥100 mg/dL or treatment of previously diagnosed diabetes | triglycerides ≥150 mg / dL or drug treatment for high triglyceride | HDL‐C <40 mg/dL for men, <50 mg/dL for women or drug treatment for low HDL‐C |
| Nyström-2015 | | Other definition standards | N/A | SBP ≥130 mm Hg and / or DBP ≥85 mm Hg and / or a history of treated hypertension | fasting glucose≥100 mg/dL or treatment of previously diagnosed diabetes | triglycerides ≥150 mg / dL or drug treatment for high triglyceride | HDL‐C <40 mg/dL for men, <50 mg/dL for women or drug treatment for low HDL‐C |
| Watanabe-2008 | | NCEP-ATPIII | N/A | SBP ≥130 mm Hg and / or DBP ≥85 mm Hg and / or a history of treated hypertension | fasting glucose≥110 mg/dL or treatment of previously diagnosed diabetes | triglycerides ≥150 mg / dL or drug treatment for high triglyceride | HDL‐C <40 mg/dL for men, <50 mg/dL for women or drug treatment for low HDL‐C |
|  |  | AHA/NHLBI |  |  | fasting glucose≥100 mg/dL or treatment of previously diagnosed diabetes |  |  |

**Abbreviations:** metabolic syndrome =MetS; National Cholesterol Education Program-Third Adult Treatment Panel = NCEP-ATPIII; American Heart Association and National Heart, Lung, and Blood Institute = AHA/NHLBI ; high-density lipoprotein cholesterol = HDL‐C; triglycerides=TG; waist circumference=WC; systolic blood pressure=SBP; diastolic blood pressure=DBP.

Supplementary Table 2：Search strategies of this meta-analysis

| Search | Query | Items found |
| --- | --- | --- |
| #1 | Metabolic syndrome [Title/Abstract] | 50719 |
| #2 | Atrial fibrillation [Title/Abstract] | 69698 |
| #3 | #1 and #2 | 237 |
